# Mendelian randomization in family data

**DOI:** 10.1186/1753-6561-3-s7-s45

**Published:** 2009-12-15

**Authors:** Nathan J Morris, Courtney Gray-McGuire, Catherine M Stein

**Affiliations:** 1 Department of Epidemiology and Biostatistics, MS 72818, Wolstein Building, 2103 Cornell Road, Case Western Reserve University, Cleveland, Ohio 44106, USA; 2 Current affiliation: Oklahoma Medical Research Foundation, 755 Research Parkway, Suite 540, Oklahoma City, Oklahoma 73104, USA

## Abstract

The phrase "mendelian randomization" has become associated with the use of genetic polymorphisms to uncover causal relationships between phenotypic variables. The statistical methods useful in mendelian randomization are known as instrumental variable techniques. We present an approach to instrumental variable estimation that is useful in family data and is robust to the use of weak instruments. We illustrate our method to measure the causal influence of low-density lipoprotein on high-density lipoprotein, body mass index, triglycerides, and systolic blood pressure. We use the Framingham Heart Study data as distributed to participants in the Genetics Analysis Workshop 16.

## Background

In epidemiological studies, establishing and measuring causal relationships is of primary importance. Unfortunately, randomization, the most important tool for unraveling causal relationships, is not generally available. Despite advances in study design and statistical adjustment, the possibility of confounding and reverse causation continues to be problematic. In recent years it has been suggested that nature itself has already performed a set of randomized experiments by assigning genes according to Mendel's laws [1]. These genes affect the function or expression levels of specific gene products, that in a cascade of cause and effect, eventually lead to human disease. By utilizing the statistical concept of an instrumental variable (IV), it may be possible to use genetics to solve some of the problems that have plagued epidemiology for decades. The goal of this approach, known as "mendelian randomization," is not to detect genetic factors of disease, but rather to use genetic factors of disease to uncover the causal relationships between phenotypes.

**Figure 1:**
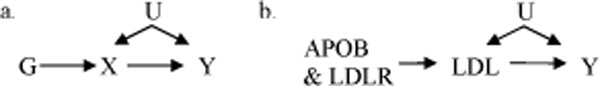
**Typical instrumental variable setup**. G is an instrument. X is a possible cause for Y. U is all unmeasured confounders.

Figure 1a shows a graphical depiction of a situation in which IV methods might be useful. Suppose that we wished to assess the relationship between *X *and *Y*. Because *U *is unmeasured, there is no way of estimating the strength of this relationship using ordinary epidemiological methodology such as linear regression. In simplistic terms, the logic behind MR is that *G *can only affect *Y *by affecting *X*. Therefore, an association between *G *and *Y *is best explained by a causal relationship between *X *and *Y*. In IV methods, we look only at how that part of *X *which is influenced by *G *affects *Y*. In order to do this we need to make at least the following three assumptions [2]:

*1. G *is not independent of *X*.

*2. G *is independent of *U*.

*3. G *is independent of *Y *given *U *and *X*.

Only Assumption 1 is testable. Assumptions 2 and 3 must be accepted or rejected using subject-specific knowledge. It is well known that there are many potential violations of these conditions when using MR. Such problems include: linkage disequilibrium, pleiotropy, population stratification, and canalization [2]. However, by careful selection of polymorphisms, many of these concerns may be minimized. A well done MR study has assumptions that cannot be tested, but these assumptions are easier to believe than those involved in a direct assessment of the relationship between *X *and *Y*.

IV techniques are well known tools in econometrics, and there is an extensive body of literature discussing the properties of various estimators. Popular methods of estimation include two-stage least-squares regression and limited information maximum-likelihood estimation [3]. These methods generally impose the assumption of linearity of the effects. Traditionally, a Wald type confidence interval of the form "estimate ± error" is used. However, a growing body of literature has shown that if the instrument is weak, this may result in confidence intervals of incorrect size [4]. A weak instrument is one which violates or comes close to violating Assumption 1 above. One approach to solving this problem is to invert tests that are robust to weak instruments [5]. We have adopted this approach below. The motivation for our approach is similar to that of G-estimation.

With few exceptions to date, discussions of MR have focused on population data. However, there is a large amount of existing family data, and there are well known advantages to family-based studies. For example, MR may provide the ability to check for mendelian errors, and it may provide protection against population stratification [6]. In this paper we suggest an approach to MR which is broadly applicable to family data.

We apply this approach to measure the causal relationship between low-density lipoprotein levels (LDL) and the variables high-density lipoprotein (HDL), triglycerides (TG), body mass index (BMI), and systolic blood pressure (SBP) in the Framingham Heart Study data as distributed to participants in the Genetic Analysis Workshop 16 (GAW16) as Problem 2. For our IV we use single-nucleotide polymorphisms (SNPs) from the 50 k data set, which are in or near the *LDLR *(OMIM 606945) and *APOB *genes (OMIM 107730), because they have direct influences on LDL. We make some additional comments in justification of our approach in the discussion.

## Methods

### Data description

We utilized the 50 k genotype data for all three cohorts of the Framingham Heart Study data. Data were obtained and used in compliance with the data use agreement and the Case Western Reserve University Institutional Review Board. We found only one SNP (rs2738457) in the *LDLR *gene in this data set. To select SNPs from *APOB *we compared the available SNPs to those analyzed in an independent sample by Benn et al. [7]. There were five SNPs shared in common between the two studies. We chose the two SNPs from Benn et al. [7] that had a *p*-value less than 0.001, appeared to be acting in an additive manner, and were shared in common with the Framingham Heart Study 50 k SNP data. These SNPs were rs1042031 and rs679899.

LDL was calculated using the Friedewald equation. In order to deal with possible heterogeneity of effect and make use of the multiple visits, each of the phenotypic observations was stratified by age at examination. The following strata were used: 0-29, 30-44, 45-60, and over 60 years of age. An approximate year of birth for each individual was calculated as the mean difference between the approximate exam date and the age at exam. All phenotype variables besides age and approximate year of birth were log-transformed for analysis in ASSOC (S.A.G.E. v5.4.1). In all analysis, the mean centered year of birth, age, and sex were used as covariates. SBP was adjusted by adding 10 to those on treatment [8].

### Statistical method

Suppose there are *K *pedigrees and *n*_*k *_individuals in each pedigree. Suppose also that **x**_*k *_and **y**_*k *_are *n*_*k *_× 1 vectors representing the variables *X *and *Y *in Figure 1. Also, G_*k *_is a *n*_*k *_× *t *matrix representing coded genotypes, and A_*k *_and B_*k *_represent matrices of covariates for **x**_*k *_and **y**_*k *_respectively. Let  and let **y **and **G **be defined analogously. We assume the following two linear structural equations:

Here, **e**_*xk *_and **e**_*yk *_are (possibly) correlated error terms. A variance-component model is assumed to describe the covariance structure . The variance-components model is described in some more detail below.

Note that *β*_*xy *_is the parameter that represents the causal effect size. Suppose that we wish to test the hypothesis H_0 _: *β*_*xy *_= *β*_*H*_. The basic approach we suggest here is to form a new trait

Combining Eq. (3) with Eqs. (2) and (1), we obtain

for suitably defined , and **e**_*rk*_. Under the model assumptions, **r**(*β*_*H*_) is independent of **G **given the covariates if and only if *β*_*xy *_= *β*_*H*_. Hence, we may test the hypothesis *H*_0 _: *β*_*xy *_= *β*_*H *_using some form of a family-based genetic association test. The test may be recomputed along an entire grid of values. Those values that are rejected at some alpha level lie outside the confidence interval. If the association between **G **and **x **is not statistically significant, then in most cases the confidence interval will cover the entire real line. For finite samples it is also possible that the confidence interval is the null set.

We have chosen to use the method implemented in the program ASSOC of S.A.G.E. (v5.4.1) to test for genetic association. This family-based test of association between markers and a continuous phenotype developed by George and Elston [9] allows for familial correlations by simultaneously estimating residual and multifactorial (polygenic, familial and marital) variance components. It is assumed that the data can be transformed via the George-Elston transformation into a multivariate distribution. ASSOC maximizes the likelihood conditional on the genotype values given the assumptions above. We supplied guesses as to the beginning and end points of the confidence interval for *β*_*xy*_. We then ran ASSOC iteratively over a grid of values for *β*_*H *_between the end points. Values for which the maximization algorithm clearly did not properly converge were not used. We used cubic spline interpolation as implemented in the R function "splinefun" to find the boundaries on the confidence interval and the point estimate. As a point estimate we used the maximum *p*-value.

We also used ASSOC to assess the causal relationships directly. We have labelled this method as "regression" below, although it is not strictly a linear regression because the familial correlation is accounted for using the same variance-component model described above. The *R*^2 ^values reported below are calculated as  where  and . Here *N*_1 _and *N*_2 _are the total number of non-missing observations on *x *and G, respectively.

## Results

As mentioned in the Introduction, we chose to look at the effect of LDL on a number of other variables (see Figure 1b). The first step in this analysis is to look at the effect of the chosen SNPs on LDL levels. There is good evidence for genetic association for all four age groups (Table 1). However, the chosen SNPs explain only a small proportion of the overall genetic variance.

**Table 1:** Genetic association with LDL

Age group (yr)	Statistical significance	*R*^2 ^for SNPs
0-29	0.005382	1.0%
30-44	8.75 × 10^-6^	0.6%
45-60	0.010954	0.3%
>60	0.011759	0.7%

There is little that can be said based on the IV analysis alone. In all cases the 95% confidence intervals are quite large. Only 1 out of 16 intervals exclude 0. It is interesting to note that in a greater-than-expected number of cases, the signs of the IV estimates and the regression estimates are the same (Table 2). The observed negative correlation between LDL and HDL is to be expected because HDL is thought to help rid the body of LDL. However, if this is the case, the causal arrow runs from HDL to LDL. The regression estimates relating HDL to LDL and TG to LDL appear to change with age. However, the regression estimates should not be overanalyzed because of potential confounders and reverse causation.

**Table 2:** Instrumental variable and direct regression estimates of effect size

Effect←Cause	Age (yr)	Regression Estimate (95% CI)^a^	IV Estimate (95% CI)^b^
BMI←LDL	0-29	**0.102 (0.074, 0.130)^c^**	0.319 (-0.143,1.454)
	30-44	**0.102 (0.085, 0.120)**	0.065 (-0.293,0.401)
	45-60	**0.054 (0.035, 0.073)**	0.452 (-0.011,1.925)
	>60	-0.008(- 0.039, 0.023)	0.156 (-0.574,1.406)
SBP←LDL	0-29	0.014 (-0.004, 0.032)	0.008 (-0.303,0.313)
	30-44	**0.024 (0.013, 0.036)**	0.026 (-0.187,0.247)
	45-60	0.013 (-0.001, 0.028)	0.369 (-0.035,2.268)
	>60	-0.009(- 0.036, 0.017)	0.085 (-0.730,0.963)
HDL←LDL	0-29	**-0.151 (-0.195, -0.107)**	-0.185(- 1.103,0.865)
	30-44	**-0.122 (-0.149, -0.096)**	-0.014(- 0.397,0.393)
	45-60	**-0.106 (-0.136, -0.077)**	0.250 (-0.552,2.800)
	>60	**0.085 (0.031, 0.139)**	-0.712(- 3.072,0.118)
TG←LDL	0-29	**0.428 (0.331, 0.524)**	**1.770 (0.500,6.230)**
	30-44	**0.313 (0.257, 0.369)**	-0.029(- 1.073,0.806)
	45-60	**0.252 (0.193, 0.311)**	0.954 (-0.767,4.892)
	>60	**-0.178 (-0.273, -0.083)**	1.038 (-0.755,7.229)

^a^The mean centered year of birth, age, and sex were used as covariates.

^b^The mean centered year of birth, age, sex and cholesterol treatment were used as covariates.

^c^Bold indicates statistical significance at *α *= 0.05.

## Discussion

It may be difficult to define exactly what is meant by "causal effect" in a system that is evolving dynamically. One-time interventions in such a system will have different effect sizes depending on the time since intervention. Eventually the system will settle back into an equilibrium state. We take the causal meaning of our results to reflect more upon the equilibrium state of the system than the instantaneous response to intervention. For example, our estimate of the causal effect of log [LDL] on log [TG] is 1.770 in the 0-29 age group. Essentially we interpret this to mean that if we could raise an individual's log [LDL] by one log [mg/dL], eventually the system would settle into a new state with log [TG] raised by about 1.770 log [mg/dL].

Every MR study should be accompanied by a biological argument that certain assumptions are met. Our argument is that polymorphisms on or near the *APOB *and *LDLR *genes will probably only affect the other phenotypes through their effect on LDL. This is because the APOB protein is one of the principal components of LDL. Similarly, LDLR is known to bind to LDL, allowing for endocytosis. Of course, the above claim could be disputed. The purpose of this study is mainly to demonstrate a statistical approach, and we do not necessarily claim that the results have a strong biological justification for Assumptions 2-3.

One concern that we believe is particularly relevant is whether the chosen markers are in linkage disequilibrium with polymorphisms that modify the function *or *the plasma levels of the LDL protein. For example, the causal effect size of LDL on HDL could differ depending on certain polymorphism in the *APOB *gene. In this case, Assumption 3 would be violated. While this would invalidate our estimates of effect size, it may not invalidate the qualitative conclusion. That is, if the confidence interval does not include 0, we still have evidence that LDL is causally related to HDL.

With regard to the selection of SNPs as instruments, we have several comments. First, we caution that in this type of study, the polymorphisms used should be justified *a priori*. Searching through a large number of possible instruments for statistical significance then performing IV analysis within the same data is not an acceptable procedure. Second, the selected polymorphisms should have biological justification as an IV. That is, it should meet Assumptions 1-3 mentioned in the introduction. Third, if there are no polymorphisms that explain a reasonably high percentage of the variance in the cause, then IVs are unlikely to be useful. Our IV estimates have very large confidence intervals. We believe this is ultimately a result of instruments that are weak predictors of LDL levels.

## Conclusion

In this paper we suggest a method for performing MR in family data. While we have chosen to use the program ASSOC to implement this method, nearly any statistical test for association between a quantitative trait and a set of polymorphisms could be used. We used ASSOC because it allows for familial correlations while using information all individuals in the family. If concerns about population stratification are raised, a more robust test such as the transmission-disequilibrium test could be used. Our approach is robust to the problem of weak instruments in the sense that it should maintain correct coverage rates. However, it will still have low power. Perhaps the biggest limitation to MR is the need for polymorphisms that explain a large percentage of the variation in a given trait.

## List of abbreviations used

BMI: Body mass index; GAW16: Genetic Analysis Workshop 16; HDL: High-density lipoprotein; IV: Instrumental variable; LDL: Low-density lipoprotein; MR: Mendelian randomization; SBP: Systolic blood pressure; SNP(s): Single-nucleotide polymorphism; TG: Triglycerides.

## Competing interests

The authors declare that they have no competing interests.

## Authors' contributions

NJM conceived of the statistical method, analyzed the data, and drafted the manuscript. CG-M helped select the traits and advised on the epidemiological aspects of the study. CMS contributed to the statistical development and writing of the paper.
